# Supplementary material for: Prevalence of early childhood caries, risk factors and nutritional status among 3-5-year-old preschool children in Kisarawe, Tanzania
Source: PLoS One. 2021 Feb 25;16(2):e0247240. doi: 10.1371/journal.pone.0247240 (PMC7906390; doi:10.1371/journal.pone.0247240)
Supplement: Attachment — Submitted filename: PONE-D-20-31062_reviewer_annotated.pdf [file pone.0247240.s002.pdf]

PLOS ONE

Prevalence of early childhood caries, risk factors and nutritional status among 3-5-year-old preschool children in Kisarawe, Tanzania

--Manuscript Draft--

**Manuscript Number:**	PONE-D-20-31062
**Article Type:**	Research Article
**Full Title:**	Prevalence of early childhood caries, risk factors and nutritional status among 3-5-year-old preschool children in Kisarawe, Tanzania
**Short Title:**	Early childhood caries, risk factors and nutritional status of preschool children, Tanzania
**Corresponding Author:**	LORNA CELIA CARNEIRO, PhD<br>Muhimbili University College of Health Sciences: Muhimbili University of Health and Allied Sciences<br>Dar es Salaam, TANZANIA, UNITED REPUBLIC OF
**Keywords:**	Early Childhood Caries, nutritional status, risk factors, sugar exposure and oral hygiene, Tanzania
**Abstract:**	Background: Early childhood dental caries (ECC) is a serious public health problem. Correlation of prevalence of ECC with risk factors and anthropometric measures will shed evidence on the link between ECC and children's anthropometric and nutritional status. The data available from Tanzania rural population regarding ECC are scarce; therefore, this study aims to assess the prevalence of ECC, risk factors and nutritional status among 3-5-year-old preschool children in Kisarawe, Tanzania and to assess its correlation with the form, visible plaque scores in upper anterior teeth, total sugar exposure, anthropometric measures, and socio demographic attributes.<br>Methods: This cross-sectional study was conducted on 831 preschool children registered in public preschools in Kisarawe District. ECC was assessed using the WHO (2013) criteria, and anthropometric measures using the WHO Child Growth Standards (2006). Structured questionnaires were completed by children's parents through an interview. Collected information on socio-demographic attributes including oral hygiene and sugar exposure to their children was analyzed.<br>Results: Only 459 children (55.2%) were recognized as caries-free. The children's mean dmft index was 2.51. Children with visible plaque were the majority (56.1%). The dmft score index was negatively and significantly associated with weight-for-age z-score [Coefficient: -0.11 (95% CI: -0.76, -0.11)] and, positively significant associated with visible plaque score index [Coefficient: 0.16 (95% CI: 0.18, 0.52)] and total sugar exposure [Coefficient: 0.19 (95% CI: 0.15, 0.38)] in the adjusted model. The prevalence of underweight was [4.2% (3.0- 5.8 95% CI)] and severe underweight was [0.2% (0.1- 0.9 95% CI)] while prevalence of stunting was [1.6% (0.9- 2.7 95% CI)] and severe stunting was [0.4% (0.1- 1.1 95% CI)].<br>Conclusion: This study demonstrated a significantly negative relationship between ECC and children anthropometric measures indicated by weight-for-age, and positive relationship with sugar exposure and poor oral hygiene indicated by visible plaque on upper anterior teeth.
**Order of Authors:**	LORNA CELIA CARNEIRO, PhD
	Tumaini Simon Ndekero
	Ray M Masumo
**Additional Information:**	
**Question**	**Response**
**Financial Disclosure**<br><br>Enter a financial disclosure statement that describes the sources of funding for the work included in this submission. Review	The funders had no role in study design, data collection and analysis, decision to publish, or preparation of the manuscript.

Powered by Editorial Manager® and ProduXion Manager® from Aries Systems Corporation

the submission guidelines for detailed requirements. View published research articles from *PLOS ONE* for specific examples.<br><br>This statement is required for submission and **will appear in the published article** if the submission is accepted. Please make sure it is accurate.<br><br>**Unfunded studies**<br>Enter: *The author(s) received no specific funding for this work.*<br><br>**Funded studies**<br>Enter a statement with the following details:<br>• Initials of the authors who received each award<br>• Grant numbers awarded to each author<br>• The full name of each funder<br>• URL of each funder website<br>• Did the sponsors or funders play any role in the study design, data collection and analysis, decision to publish, or preparation of the manuscript?<br>• **NO** - Include this sentence at the end of your statement: *The funders had no role in study design, data collection and analysis, decision to publish, or preparation of the manuscript.*<br>• **YES** - Specify the role(s) played.<br><br>* typeset	
**Competing Interests**<br><br>Use the instructions below to enter a competing interest statement for this submission. On behalf of all authors, disclose any competing interests that could be perceived to bias this work—acknowledging all financial support and any other relevant financial or non-financial competing interests.<br><br>This statement **will appear in the published article** if the submission is accepted. Please make sure it is accurate. View published research articles from *PLOS ONE* for specific examples.	The authors have declared that no competing interests exist.

**NO authors have competing interests**<br><br>Enter: *The authors have declared that no competing interests exist.*<br><br>**Authors with competing interests**<br><br>Enter competing interest details beginning with this statement:<br><br>*I have read the journal's policy and the authors of this manuscript have the following competing interests: [insert competing interests here]*<br><br>* typeset	
**Ethics Statement**<br><br>Enter an ethics statement for this submission. This statement is required if the study involved:<br><br>• Human participants<br>• Human specimens or tissue<br>• Vertebrate animals or cephalopods<br>• Vertebrate embryos or tissues<br>• Field research<br><br>Write "N/A" if the submission does not require an ethics statement.<br><br>General guidance is provided below. Consult the submission guidelines for detailed instructions. **Make sure that all information entered here is included in the Methods section of the manuscript.**	Approval of the study was granted by the Senate Research and Publication Committee of the Muhimbili University of Health and Allied Sciences - (Ref. No. DA282/298/01.C) written consent was obtained from parents of children

Powered by Editorial Manager® and ProduXion Manager® from Aries Systems Corporation

**Format for specific study types**<br><br>**Human Subject Research (involving human participants and/or tissue)**<br>• Give the name of the institutional review board or ethics committee that approved the study<br>• Include the approval number and/or a statement indicating approval of this research<br>• Indicate the form of consent obtained (written/oral) or the reason that consent was not obtained (e.g. the data were analyzed anonymously)<br><br>**Animal Research (involving vertebrate animals, embryos or tissues)**<br>• Provide the name of the Institutional Animal Care and Use Committee (IACUC) or other relevant ethics board that reviewed the study protocol, and indicate whether they approved this research or granted a formal waiver of ethical approval<br>• Include an approval number if one was obtained<br>• If the study involved *non-human primates*, add *additional details* about animal welfare and steps taken to ameliorate suffering<br>• If anesthesia, euthanasia, or any kind of animal sacrifice is part of the study, include briefly which substances and/or methods were applied<br><br>**Field Research**<br><br>Include the following details if this study involves the collection of plant, animal, or other materials from a natural setting:<br>• Field permit number<br>• Name of the institution or relevant body that granted permission	
**Data Availability**<br><br>Authors are required to make all data underlying the findings described fully available, without restriction, and from the time of publication. PLOS allows rare exceptions to address legal and ethical concerns. See the PLOS Data Policy and FAQ for detailed information.	Yes - all data are fully available without restriction

A Data Availability Statement describing where the data can be found is required at submission. Your answers to this question constitute the Data Availability Statement and **will be published in the article**, if accepted.<br><br>**Important:** Stating 'data available on request from the author' is not sufficient. If your data are only available upon request, select 'No' for the first question and explain your exceptional situation in the text box.<br><br>Do the authors confirm that all data underlying the findings described in their manuscript are fully available without restriction?	
**Describe where the data may be found in full sentences. If you are copying our sample text, replace any instances of XXX with the appropriate details.**<br><br>• If the data are **held or will be held in a public repository**, include URLs, accession numbers or DOIs. If this information will only be available after acceptance, indicate this by ticking the box below. For example: *All XXX files are available from the XXX database (accession number(s) XXX, XXX.)*.<br>• If the data are all contained **within the manuscript and/or Supporting Information files**, enter the following: *All relevant data are within the manuscript and its Supporting Information files.*<br>• If neither of these applies but you are able to provide **details of access elsewhere**, with or without limitations, please do so. For example:<br><br>*Data cannot be shared publicly because of [XXX]. Data are available from the XXX Institutional Data Access / Ethics Committee (contact via XXX) for researchers who meet the criteria for access to confidential data.*<br><br>*The data underlying the results presented in the study are available from (include the name of the third party*	All data files are available from the spss database

and contact information or URL).<br>• This text is appropriate if the data are owned by a third party and authors do not have permission to share the data.<br><br>* typeset	
Additional data availability information:	Tick here if your circumstances are not covered by the questions above and you need the journal's help to make your data available.

Powered by Editorial Manager® and ProduXion Manager® from Aries Systems Corporation

Manuscript

Click here to access/download;Manuscript;ECC and nutritional statatus- 2nd Oct 2020 -upload version.doc

Title

Prevalence of early childhood caries, risk factors and nutritional status among 3-5-year-old preschool children in Kisarawe, Tanzania.

Authors

Tumaini S. Ndekero[1], Lorna C. Carneiro[1*], Ray M. Masumo[2]

Affiliations

[1] Department of Restorative Dentistry, Muhimbili University of Health and Allied Sciences, Dar es salaam, Tanzania.

[2] Oral Health Section, Ministry of Health, Community Development, Gender, Elderly and Children, Dodoma, Tanzania.

*Corresponding author:

Email: carneiro2@hotmail.com (LCC)

Abstract

Background: Early childhood dental caries (ECC) is a serious public health problem. Correlation of prevalence of ECC with risk factors and anthropometric measures will shed evidence on the link between ECC and children's anthropometric and nutritional status. The data available from Tanzania rural population regarding ECC are scarce; therefore, this study aims to assess the prevalence of ECC, risk factors and nutritional status among 3-5-year-old preschool children in Kisarawe, Tanzania and to assess its correlation with the form, visible plaque scores in upper anterior teeth, total sugar exposure, anthropometric measures, and socio demographic attributes.

Methods: This cross-sectional study was conducted on 831 preschool children registered in public preschools in Kisarawe District. ECC was assessed using the WHO (2013) criteria, and anthropometric measures using the WHO Child Growth Standards (2006). Structured questionnaires were completed by children's parents through an interview. Collected information on socio-demographic attributes including oral hygiene and sugar exposure to their children was analyzed.

Results: Only 459 children (55.2%) were recognized as caries-free. The children's mean dmft index was 2.51. Children with visible plaque were the majority (56.1%). The dmft score index was negatively and significantly associated with weight-for-age z-score [Coefficient: -0.11 (95% CI: -0.76, -0.11)] and, positively significant associated with visible plaque score index [Coefficient: 0.16 (95% CI: 0.18, 0.52)] and total sugar exposure [Coefficient: 0.19 (95% CI: 0.15, 0.38)] in the adjusted model. The prevalence of underweight was [4.2% (3.0- 5.8 95% CI)]

and severe underweight was [0.2% (0.1- 0.9 95% CI)] while prevalence of stunting was [1.6% (0.9- 2.7 95% CI)] and severe stunting was [0.4% (0.1- 1.1 95% CI)].

Conclusion: This study demonstrated a significantly negative relationship between ECC and children anthropometric measures indicated by weight-for-age, and positive relationship with sugar exposure and poor oral hygiene indicated by visible plaque on upper anterior teeth.

Key words: Early Childhood Caries, nutritional status, risk factors, sugar exposure and oral hygiene, Tanzania.

Introduction

The World Health Organization (WHO) data suggest that the prevalence of dental caries has declined at the end of the previous and in the first decade of the present century [1]. Despite this considerable decline of a 90 % reduction in DMFT for 12-year-olds in Western Europe and the USA, caries still affects 60–90 % of the children throughout the world [1]. Although preventable, Early Childhood Caries (ECC) to 3-5 years old is a major dental public health concern not only in most European countries and the USA but also in disadvantaged communities in both developing and industrialized countries [1,2].

While some studies have evaluated and categorized the risk factors of ECC, to be related to sociodemographic factors, dietary factors and oral hygiene factors [3–5] other studies report familial socioeconomic background, lack of parental education, and lack of access to dental care [2] to be contributing factors for the high prevalence of ECC. However, the degree to which different risk factors are associated with ECC remains unclear.

ECC has been associated with other health problems, ranging from local pain, infections, abscesses, leading to difficulty in chewing, malnutrition and difficulty in sleeping [6]. Furthermore, the associated pain from dental caries has a negative impact on children’s emotional status, sleep patterns, and ability to learn or perform their usual activities. Preschool children with ECC, if left untreated, might experience dental pain leading to avoidance of certain types of foods which might interfere adversely with their nutritional status [7,8].

Preschool children need healthy teeth to chew and masticate foods, when they graduate from the weaning diet [9,10]. According to the UNICEF multifactorial model of 1990, sufficient dietary intake is among the immediate determinants of children's nutritional status.

Studies conducted among preschool children have documented the link between ECC with the detrimental impacts on the growth and development of a child although others have shown weak or no relationship between nutritional status and ECC [11]. Studies conducted by Clarke and colleagues in 2006 [12] and Wasunna in 2012 [13] reported differences in the percentiles of weights of under five years old preschool children with dental caries and without dental caries. Furthermore, studies by Filstrup and colleagues in 2003 [14] documented that pain, discomfort, and irritability attributed to caries have been associated with reduced food intake, disturbed sleeping habits, and impaired secretion of growth hormones. Shreds of evidence on the link between untreated ECC and children’s nutritional status remain scanty, controversial, and non-conclusive [15,16].

In Africa the reported prevalence of ECC ranged between 47%, 58%, and 63% among 3, 4, and 5 years old in South Africa [17] to 60% of girls and 52% of boys aged 3-5 years old in Khartoum-Sudan [18]. Similar findings were also documented in East African countries on the high prevalence of ECC among preschool children. Kiwanuka and colleagues in Uganda in 2004

found that the prevalence of ECC among children aged 3–5-years old was 56% in Kampala and 64% in Nakawa [19]. Njoroge and colleagues in Kenya in 2010 reported the prevalence of ECC among preschool children aged 3-5 years old was 59.5% and, the highest component (95%) was the decayed component [20]. In Tanzania, Rwakatema and Ng'ang'a in 2010, reported prevalence of ECC among 3-5 years old preschool to 30.1% in Moshi Municipality with the highest component of 87% as the decayed [21]. Masumo and colleagues in their study of preschool children aged 4–6 years in Kisarawe documented that one third (30.2%) of participating preschool children had caries experience [11]. Furthermore, Mishu and colleagues in 2013 documented that pain and dysfunction experienced by ECC affected the chewing of food leading to practice food selection [16]. Practice of food selection can affect the quantity and quality consumed resulting in under nutrition or over nutrition for those who select easy to eat fast foods [22]. The prevalence of stunting among preschool children as reported by the Tanzania National Nutrition Surveys (TNNS) decreased significantly from 34.7% in 2014 [23] to 31.8% in 2018 [24]. The significant decrease in the prevalence of stunting was observed in seven regions of Tanzania Mainland including the Pwani region [24]. Further the TNNS of 2018 acknowledged that the newly recorded prevalence of underweight of 14.6% (13.9-15.3 95% CI) was significantly higher as compared to the recorded prevalence in TNNS 2014 (13.4%; $p<0.05$) [23,24].

There is a lack of information on the association between ECC, risk factors and nutritional status among children in Tanzania due to limited studies. The present study aimed to assess the prevalence of ECC in preschool children of 3–5-years in Kisarawe District and the associated risk factors and nutritional status by determining the visible plaque scores in upper anterior teeth, total sugar exposure, anthropometric measures, and socio demographic attributes. This study was

able to determine the relationship between ECC, risk factors and nutritional status of the preschool children in Kisarawe, Tanzania.

Materials and methods

A cross-sectional study was conducted in Kisarawe district, one of the 6 districts in the Pwani Region of Tanzania. The district was conveniently chosen due to its rural (population of 84,174) and semi-urban (population of 17,424) characteristics [25]. The arrangement of the formal education in Tanzania institutes 2 years of pre-primary education, 7 years of primary education, 4 years of ordinary secondary education, 2 years of advanced secondary education, and a minimum of 3 years of university education [26]. The official school-age of pre-primary education is 3 -6 years [26]. Kisarawe district is administratively divided into 15 wards which have a semi-urban and rural population: Cholesamvula, Kibuta, Kiluvya, Kisarawe, Kurui, Mafizi, Maneromango, Marui, Marumbo, Masaki, Msanga, Msimbu, Mzenga, Vihingo, and Vikumbulu.

The district is home to about 108,398 people, out of whom 3.1% is 3 years old, 3.2 % is 4 years old and 3.0% is 5 years old [25] and, had eighty-three registered preschools at the time of the study [26]. Ethical approval for this study was granted by the Senate Research and Publication Committee of the Muhimbili University of Health and Allied Sciences in Tanzania (Ref. No. DA282/298/01.C). Further permission was granted by the District Executive Director and District Education Officer (President's Office, Regional Administrative and Local Government-Tanzania). With assistance of these offices one preschool from each ward was randomly selected. Respective Head-teachers were informed of the study and were requested to provide each child registered for preschool with a consent form for parents/guardians to sign and a letter

requesting them to escort their child to school on scheduled days. The total sample size of 831 preschool children was estimated by assuming the proportion of weight and height, and ECC (outcome) among preschool children in Tanzania was 50%, with a margin error of 5%, confidence level of 95% and an assumed design effect of 2. Another 5% was added to the estimated sample size to account for non-response. Included in the study were children who were present with their parents or guardians and with signed consent to participate in the research.

Interviews

A structured questionnaire was translated in several steps; from English into Kiswahili by bi-lingual Kiswahili/English professionals, and then translated back to English by independent translators. Kiswahili is the national language spoken proficiently by almost 99% of the Tanzanian population and the reason for its use. Project professionals in the field reviewed the structured questionnaire for semantic, experiential, and conceptual equivalence to the original version. Sensitivity to culture and selection of appropriate words were considered.

Trained researchers (TS and LC) interviewed parent or guardian accompanying each child using the structured questionnaire. Information collected included sociodemographic factors, dietary habits, and oral health behavior of the children. Parent/guardian level of education and employment was also sought.

Demographic factors of preschool children were assessed in terms age, sex, and area of residency. Parents' reported children's behaviors on consumption of sugar snacks and drinks (biscuits or cake; candy or chocolate and; sweetened milk or soft drinks) being guided by the following questions: Does your child consume sugary food in between meals (0= No, 1= Yes); How many times a day does your child consume sugary food (0= less than 5 times a day, 1= more than 5 times a day); In the past 7 days did your child consume sugary foods (0= never, 1=

one day, 2= two to four days, 3= five days and more); What is the amount of sugar teaspoon usually consumed by your child in a day (0= No, 1= one to two teaspoon, 2= more than two teaspoon); Does your child ever report not being hungry during meal time cause of eating sugary food (0= No, 1= Yes); Do you often sweeten your child's milky food with sugar (0= No, 1= Yes); Do you often sweeten your child starchy food (e.g. porridge) with sugar (0= No, 1= Yes); Does your child consume frequent fruit juices or soda (0= No, 1= Yes) and; Does your child consume sweets (e.g. biscuits, cake, candy or chocolate) (0= No, 1= Yes). Total sugar consumption was calculated based on the overall intake of frequency. Scores of snacks and drinks were then summed to produce an overall score (range 2- 23). The overall score was used to indicate the daily frequency of intake of sugars for each participant. In addition parents' reported children's daily behavior of consuming fresh fruits and vegetables: Does your child consume fruits (0= no, 1= once a day, 2= twice a day, 3= thrice a day, 4= more than 3 times a day) and; Does your child consume vegetables (0= no, 1= once a day, 2= twice a day, 3= more than 2 times a day). Dummy variables were summarized (range 0- 7) and used as a continuous variable. Other oral health behaviors were assessed by asking parents to report on: Do you assist your child to brush (0= No, 1= Yes); Do you use fluoride toothpaste while brushing your child (0= No, 1= Yes) and; how frequent does your child brush (0= no, 1= once a day, 2= twice a day, 3= thrice a day, 4= more than 3 times a day) and then summarized into (0= No, 1= Yes). Parent/guardian was requested to provide information on level of education (0=primary and lower level of education, 1=secondary and higher level of education) and employment (0= not employed, 1= employed) of both parents/guardians of the child.

Clinical examination

Calibration exercises for the examiners with respect to presence/absence of visible plaque [27] and early childhood caries were carried out according to the guidelines published by the British Association of the Study of Community Dentistry (BASCD) [28].

Children were clinically examined using a dental mirror and natural light by calibrated dentists (TN and LC) while in a knee-to-knee position. Upper anterior teeth; incisors, lateral incisors and canines were examined visually for presence of plaque which was recorded on the basis of absence of visible plaque = 0 or presence of visible plaque = 1. Dummy variables (0 = No, 1 = Yes) were summarized (range 0 – 6) and dichotomized into children with a count of a plaque score of 0 as having "good oral hygiene" while children with plaque score of one or more were regarded as having "poor oral hygiene".

Following plaque assessment, visual inspection of children's deciduous teeth for ECC was done after wiping and drying using a piece of sterile gauze. With the aid of a disposable dental mirror, absence/presence of a carious lesion was recorded in the recommended WHO Oral Health Assessment Form for Children, 2013 [29]. A score of 0 denoted absence of any visible carious lesion while a score of 1 denoted presence of visible carious lesion. Missing and filled teeth were given a score of 1. In the present analysis, dmft was used as a count variable following dichotomizing into caries free (dmft = 0) or with caries experience (dmft =1).

Nutritional status

Nutritional status among preschool children was assessed by anthropometric measures based on the WHO Growth Standards 2006 [30]. A trained registered nurse from the Muhimbili National Hospital measured and recorded the weight and height of each child. Preschool children were asked to remove their shoes before measurement of weight and height were taken using the Seca

weight and height measuring machine (Model 786, Mechanical column scale with large round dial and stadiometer, CE 0123, Vogel and Halke, Germany). Based on the WHO Growth Standards 2006, weight, height and age measures were converted to z-scores, namely weight-for-age, weight-for-height, and height-for-age [30]. The Z-score was used because of it recognition as the best system for analysis and presentation of anthropometric data for preschool children and because of its advantages compared to the other methods [31].

Reliability of ECC and anthropometric measures

Prior to the fieldwork, two trained dentists (TN and LC) were calibrated for scoring of ECC lesions in preschool children. Calibration was performed using images of different clinical situations on two separate occasions with a one-week interval between sessions. The minimum intra-examiner agreement was 0.83 and the minimum inter-examiner agreement was 0.78. During the fieldwork, duplicate examinations one week apart were performed with 74 preschool children randomly chosen. Intra examiner reliability in terms of Cohen's kappa ranged from 0.81 to 0.89, respectively.

The person who did anthropometric measurements was calibrated prior to the study with an agreement was 0.85. Being blinded to the oral health status, weight and height of participants were measured with a weight and height scale to the nearest 0.5 kilograms and 0.5 cm respectively. The equipment was recalibrated daily prior to the procedure. The level of intra-examiner agreement (Cohen's kappa) of 0.72, was considered substantial.

Statistical analysis

The collected data were entered into the Epi Info version 3.5.4 and ENA Software and further analysis in Predictive Analytics Software, IBM SPSS Statistics version 20. To determine the mean of the children's dmft index and the prevalence of the children with ECC, Pearson Chi-square and One-way ANOVA was used to estimate the relationship between demographic factors (age, sex, and residency) and oral health behaviors (assist the child to brush, child use toothpaste while brushing and, frequency of child tooth brushing). Further, the description of anthropometric measurement (actual weight and height, and z-score for weight-for-age, height-for-age and weight-for-height), visible plaque score index, sugar consumption index, fresh fruit consumption index, and ECC (dmft index) among preschool children was determined.

To control the effect of possible confounding factors, all variables were entered into multiple logistic regression and Poisson regression models with the dmft >1 and, dmft index as dependent variables. Moreover, a simple linear regression model with the children's dmft index as the dependent variable was constructed to explore the relationship with weight (in Kg), height (in cm), age (in months), weight-for-age z-score (WAZ), height-for-age z-score (HAZ), weight-for-height z-score (WHZ), Visible plaque score index, sugar consumption and, fresh fruit consumption. An alpha level of 0.05 was considered as statistical significance. The multilevel linear analysis was further constructed to explore the association between dmft index (dependent variable) and, weight-for-age z-score, height-for-age z-score, weight-for-height z-score (independent variables), adjusted with all variables associated with either dmft >1 or dmft index (visible plaque score index, sugar consumption, residence, mothers working status, assist child to brush and, child use toothpaste during brush). The same sets of models were used to assess the bidirectional association between dmft index (independent variable) and weight-for-age z-score, height-for-age z-score, weight-for-height z-score (dependent variables).

Results

Most parents of preschool children (N=860) invited to the study consented to participate (response rate= 97%). Although 80.4% (n=668) and 86.3% (n= 717) of preschool fathers and mothers had primary education, more than ninety percent of fathers and mothers (n= 746 and n= 795) were not employed respectively. The mean age of the participants was 50.34 (±8.41) months with almost equal male/female ratio [male= 48.3% (n= 401) and female= 51.7 (n= 430)]. Mother parents 67.9% (n=564) reported not to assist their children tooth brushing and 81.0% (n= 673) reported their children to use toothpaste while brushing. However only 23.1% (n= 192) reported their children to brush more than once in a day.

Children's oral health status

According to the clinical oral examination, prevalence of ECC was 44.8%. Children with one to four, and more than five decayed teeth were 47.7% (n= 219), and 21.8% (n= 101), respectively. The dmft of 2.51 was contributed to mainly by the decayed component. More than half [56.1% (n= 466)] of the participating preschool children had visible plaque on buccal surfaces of upper anterior teeth. Of the 56.1% children with visible plaque, 11.8% (n= 55), 37.8% (n= 176), and 50.4% (n= 235) had one, two, and more than two teeth with visible plaque, respectively.

Results of anthropometric measures

Figure 1 shows the distribution of Weight-for-Age of preschool children aged 3 to 5 years in Kisarawe is shifted to the left but still following the WHO standard natural distribution of reference population when WHO flags are applied with mean z-score -0.39 ± 1.06 SD.

Figur[illegible] Weight-for-Age z-score (WHO 2006)

As shown in Table 1, the prevalence of underweight (<-2 z-score) was [4.2% (3.0- 5.8 95% CI)] and, severe underweight (<-3 z-score) was [0.2% (0.1- 0.9 95% CI)].

Table 1- Simple linear regression analysis dmft index with anthropometric measures, visible plaque score index, sugar consumption index and fresh fruit consumption index

No	Variable	Mean ± SD	Coefficient (95% [illegible])
1	Weight (in Kg)	15.82 ± 2.10	-0.03 (-0.15, 0.07)
2	Height (in cm)	106.6 ± 6.67	-0.02 (-0.05, 0.03)
3	Age (in months)	50.34 ± 8.407	-0.03 (-0.04, 0.02)
4	Weight-for-age z-score (WAZ)<br>• Prevalence of underweight (<-2 z-score) = 4.2% (3.0- 5.8 95% CI)<br>• Prevalence of moderate underweight (<-2 z-score and >=-3 z-score) = 4.0% (2.8- 5.5 95% CI)<br>• Prevalence of severe underweight (<-3 z-score) = 0.2% (0.1- 0.9 95% CI)	-0.39 ± 1.06	-0.07 (-0.61, 0.003)*
5	Height-for-age z-score (HAZ)<br>• Prevalence of stunting (<-2 z-score) = 1.6% (0.9- 2.7 95% CI)<br>• Prevalence of moderate stunting (<-2 z-score and >=-3 z-score) = 1.2% (0.7- 2.2 95% CI)<br>• Prevalence of severe stunting (<-3 z-score) = 0.4% (0.1- 1.1 95% CI)	0.57 ± 1.08	-0.03 (-0.45, 0.19)
6	Weight-for-height z-score (WHZ)<br>• Prevalence of global acute malnutrition (<-2 z-score and/or oedema) = 29.8% (26.8-33.0 95% CI)<br>• Prevalence of moderate acute malnutrition (<-2 z-score and >=-3 z-score, no oedema) = 24.7% (21.9-27.8 95% CI)<br>• Prevalence of severe acute malnutrition (<-3 z-score and/or oedema) = 5.1% (3.8- 6.8 95% CI)	-1.18±1.45	-0.03 (-0.35, 0.15)
7	Visible plaque score index	1.40 ± 1.38	0.18 (0.22, 0.56)***
8	Sugar consumption	15.45 ± 2.12	0.17 (0.13, 0.36)***
9	Fresh fruit consumption	2.83 ± 1.08	0.02 (-0.19, 0.27)
	*** p<0.01<br>*p<0.05		

The Figure 2 shows that the distribution of Height-for-Age of the assessed preschool children in Kisarawe was shifted to the right and was flatter as compared to the WHO standard normal distribution of reference population even when WHO flags are applied. The mean HAZ was 0.57 ± 1.08. The distribution was flattened may contributed by difficulties encountered during data collection for age estimation.

Figure 2: Height-for-Age z-score (WHO 2006)

As shown in Table 1, the prevalence of stunting (<-2 z-score) was [1.6% (0.9- 2.7 95% CI)] and, severe stunting (<-3 z-score) was [0.4% (0.1- 1.1 95% CI)]. Figure 3 shows that the distribution of Weight-for-Height is shifted to the left but still following the WHO standard normal distribution of reference population, with mean WHZ -1.18±1.45 SD. The standard deviation indicates difficulties encountered during data collection for age estimation during data collection.

Figure 3: Weight-for-Height z-score (WHO 2006)

As shown in Table 1, the prevalence of global acute malnutrition (<-2 z-score and/or oedema) was [29.8% (26.8-33.0 95% CI)] and, severe acute malnutrition (<-3 z-score and/or oedema) was [5.1% (3.8- 6.8 95% CI)].

Univariate analysis of risk factors associated with dmft ≥1 and dmft score index

In univariate analysis as presented in Table 2, chi-square test showed that children whose residency are semi-rural and having visible plaque on upper anterior teeth were significant associated with the prevalence of ECC (dmft ≥1) both p< 0.001.

Further**,** the one-way between groups analysis of variance (ANOVA) conducted to explore the impact of various risk factors on the mean score of dmft index showed a statistically significant difference at the p < 0.001 level in dmft index scores at two residency groups F (1, 616) = 6.37, p

< 0.001. Despite reaching statistical significance, the actual difference in mean scores between groups was quite small. The effect size, calculated using eta squared, was 0.01.

Table 2 – Univariate and multivariate analysis of different factors associated with dental caries status in deciduous tooth (n=831)

		**Univariate analysis**		**Multivariate analysis**	
		**dmft ≥1**	**dmft index**	**dmft ≥1**	**dmft index**
**Variables**	**Categories**	[1a] **Caries prevalence % (n)**	[1b] **F- test (df)**	[1c] **OR, 95% CI**	[1d] **RR, 95% CI**
Child sex	Male	50.3 (187)	0.85 (1)	1	1.08 (0.98, 1.19)
	Female	49.7 (185)		1.11 (0.85, 1.46)	1
Child age	3 years	3.2 (12)	0.41 (2)	1	1.12 (0.88, 1.42)
	4 years	18.8 (70)		1.48 (0.65, 3.37)	0.92 (0.81, 1.05)
	5 years	78.0 (290)		1.02 (0.47, 2.18)	1
Residence	Rural	84.7 (315)	6.37 (1)***	1	1. 34 (1.15, 1.56) ***
	Semi-rural	15.3 (57)***		0.55 (0.39, 0.79)***	1
Mothers level of education	Informal and Primary education	86.6 (322)	2.48 (1)	1	1.034 (0.8, 1.25)
	Secondary education and above	13.4 (50)		0.96 (0.64, 1.43)	1
Fathers level of education	Informal and Primary education	79.8 (295)	1.81 (1)	1	1.11 (0.95, 1.29)
	Secondary education and above	20.2 (75)		0.72 (1.07, 1.58)	1
Mothers working condition	Unemployed	96.2 (358)	4.03 (1)*	1	1.65 (1.19, 2.27)***
	Employed	3.8 (14)		0.78 (0.39, 1.53)	1
Fathers working condition	Unemployed	90.1 (335)	0.92 (1)	1	0.95 (0.78, 1.15)
	Employed	9.9 (37)		0.95 (0.60, 1.49)	1
Do you assist your child to brush?	No	64.5 (240)	0.50 (1)	1	0.85 (0.77, 0.95)***
	Yes	35.5 (132)		1.32 (0.98, 1.77)	1
Does your child use toothpaste while brushing?	No	18.3 (68)	3.23 (1)	1	1.18 (1.04, 1.34)***
	Yes	81.7 (304)		1.09 (0.77, 1.55)	1
Frequent child tooth brushing	Not every day	3.0 (11)	0.10 (2)	1	1.10 (0.79, 1.54)
	Once a day	73.9 (275)		1.04 (0.45, 2.45)	1.11 (0.98, 1.25)
	Twice or more a day	23.1 (86)		1.01 (0.73, 1.39)	1
Visible plaques dichotomous score	No	29.6 (110)***	16.08 (1)	1	0.69 (0.58, 0.73)***
	Yes	70.4 (262)		2.97 (2.23, 3.96)***	1

[1a] *Pearson Chi-square test*
[1b] *One Way Anova*
[1c] *Logistic regression*
[1d] *Poisson regression*

*** p<0.01
*p<0.05

As shown Tabl[illegible] a simple linear regression was carried out to test if weight (in Kg), height (in cm), Age (in months), weight-for-age z-score, height-for-age z-score, weight-for-height z-score, visible plaque score index, sugar consumption scores and, fresh fruit consumption scores significantly predicted dmft score index. The results of the regression indicated that the dmft score index was negatively and significantly associated with weight-for-age z-score (Coefficient: -0.07; 95% CI: -0.61, 0.00) in the unadjusted model. Moreover, there were significant increases in visible plaque scores index and sugar consumption scores with regression coefficient of 0.18 (95% CI: 0.22, 0.56) and 0.17 (95% CI: 0.13, 0.36), respectively.

Multivariate Logistic regression, Poisson regression and Linear regression analysis of risk factors associated with dmft ≥1 and dmft score index

As shown in Table 2, lower prevalence of ECC (i.e. dmft ≥1) had been associated with children whose residency where from semi-rural [adjusted OR 0.55 (95% CI: 0.39, 0.79)] and, higher prevalence from children with presence of visible dental plaque [adjusted OR 2.97 (95% CI: 2.23, 3.96)]. Poisson regression were employed to rule out the risk of losing precision when dmft ≥1 employed in the multiple variable logistic regression analysis. The dmft scores index shows significant association with residence [adjusted RR 1. 34 (95% CI: 1.15, 1.56)], mothers working condition [adjusted RR 1.65 (95% CI: 1.19, 2.27)], do you assist your child to brush? [Adjusted RR 0.85 (95% CI: 0.77, 0.95)], does your child use toothpaste while brushing? [Adjusted RR 1.18 (95% CI: 1.04, 1.34)] and, presence of visible plaques [adjusted 0.69 (95% CI: 0.58, 0.73)].

As shown in Table 3, a multilevel linear analysis to explore the association between change dmft score index and weight-for-age z-score adjusted with visible plaque score index, sugar consumption, residence, mothers working status, assist child to brush and, child use toothpaste during brush revealed that: The dmft score index was negatively and significantly associated

with weight-for-age z-score [Coefficient: -0.11 (95% CI: -0.76, -0.11)] and, positively significant associated with visible plaque score index [Coefficient: 0.16 (95% CI: 0.18, 0.52)] and sugar consumption score [Coefficient: 0.19 (95% CI: 0.15, 0.38)] in the adjusted model. However, dmft score index was not statistically significant with height-for age z-score and weight for height z-score.

Table 3. Multilevel linear analysis of factors associated with dmft score index

	Dmft score index		
**Variable**	**Coefficient (95% CI)**	**Coefficient (95% CI)**	**Coefficient (95% CI)**
Weight for age z-score	-0.11 (-0.76, -0.11) ***		
Height for age z-score		-0.07 (-0.59, 0.05)	
Weight for height z-score			-0.05(-0.41, 0.08)
Visible plaque index	0.16 (0.18, 0.52) ***	0.16 (0.17, 0.52) ***	0.16 (0.18, 0.52)***
Sugar consumption	0.19 (0.15, 0.38) ***	0.18 (0.15, 0.37) ***	0.18 (0.14, 0.37)***
Residence	-0.06 (-1.10, 0.15)	-0.07 (-1.14, 0.16)	-0.07 (-1.16, 0.09)
Mothers working status	-0.07 (-2.01, 0.09)	-0.07 (-2.01, 0.11)	-0.07 (-2.03, 0.09)
Do you assist your child to brush	-0.06 (-0.10, 0.92)	0.07 (-0.09, 0.94)	0.07 (-0.08, 0.95)
Does your child use toothpaste during brush	-0.08 (-1.26, 0.01)*	-0.08 (-1.24, 0.04)	-0.07 (-1.19, 0.09)
*** p<0.01<br>*p<0.05			

In Table 4 we further explored the bilateral relation of weight-for-age z-score, height-for age z-score and, weight for height z-score (as dependent variables) and dmft score index (as dependent variable) adjusted with visible plaque score index, sugar consumption, residence, mothers working status, assist child to brush and, child use toothpaste during brushing revealed that: weight-for-age z-score were still negatively and significantly associated with dmft score index

[Coefficient: -0.11(95% CI: -0.05, -0.01)]. However, height-for age z-score and, weight for height z-score were not statistically significant with dmft score index.

Table 4. Multilevel linear analysis of factors associated with changes in Weight for age z-score, Height for age z-score and Weight for height z-score

	**Weight for age z-score**	**Height for age z-score**	**Weight for height z-score**
**Variable**	**Coefficient (95% CI)**	**Coefficient (95% CI)**	**Coefficient (95% CI)**
dmft index	-0.11(-0.05, -0.01)***	-0.07 (-0.04, 0.003)	-0.06 (-0.04, 0.01)
Visible plaque index	0.06 (-0.01, 0.07)	0.04 (-0.02, 0.06)	0.05 (-0.02, 0.09)
Sugar consumption	0.09 (0.01, 0.06)*	0.09 (0.003, 0.06)*	0.05 (-0.02, 0.06)
Residence	0.08 (0.01, 0.32)*	0.07 (-0.02, 0.30)	0.05 (-0.07, 0.34)
Mothers working status	0.01 (-0.23, 0.29)	0.03 (-0.16, 0.37)	0.02 (-0.25, 0.44)
Do you assist your child to brush	-0.07 (-0.24, 0.02)	-0.09 (-0.27, -0.02)*	-0.07 (-031, 0.02)
Does your child use toothpaste during brush	-0.08 (-0.30, 0.02)	-0.06 (-0.28, 0.04)	0.04 (-0.11, 0.31)
*** p<0.01<br>*p<0.05			

Discussion

This population based cross-sectional study aims to described the eff[illegible] of ECC on nutritional status of preschool children in the Kisarawe District of Pwani Region, Tanzania. There is a growing body of evidence that suggests significant decline of dental caries among 12-year-olds in most developed countries [32], however, ECC is still affecting 60–90 % of preschool children throughout the world [1]. The most important from a general health and well-being point of view is that among preschool children, over 90% of ECC remains untreated accompanied with

discomfort or toothache affecting young children’s growth and well-being [10]. There is dearth of information documented on how untreated carious lesions does affect the growth and wellbeing of millions of young children in Sub- Saharan African countries including Tanzania. This lack of information has been observed despite the Regional strategy on Oral health 2016-2025 that emphasized on shifting the focus of dental research from the causes of the dental diseases to how dental diseases affect general health [33].

In this study, we revealed that the prevalence of ECC was 44.8% among preschool children aged 3-5 years old in Kisarawe District in Tanzania which is considerable higher as compared to the prevalence of 30.1% that was documented in the study of Rwakatema and Ng'ang'a among 3-5 years old in Moshi Municipality, Tanzania [21], and slightly similar to prevalence of 48.6% documented among 3–5 years old from a rural community of Uganda [34]. However, the prevalence of ECC reported in this study is still higher as compared to the prevalence documented by Nobile and colleagues in Southern Italy [35]. Furthermore, the mean dmft index score in the children of this study was slightly similar to that documented by Musinguzi and colleagues among preschool children in the rural community [34], and considerably higher than that of developed countries [36]. The documented low prevalence of ECC in developed countries is much contributed by the implementation of effective prevention programs, which should be considered in developing countries. Increased use of fluoride toothpaste was the most probable reasons for decrease in ECC in most developed countries [1]. Evidences have also shown that application of 5% sodium fluoride varnish can remineralize early enamel caries in children [37] and, using silver diamine fluoride solution at 38% can arrest active dentine caries when applied biannually [37].

Sufficient oral hygiene is one of the prerequisites for preventing ECC to preschool children. However, the majority of the preschool children in the present study unveiled poor oral hygiene, characterized by the presence of clinically visible plaque on upper anterior teeth. Different studies have further documented on association between ECC and supervision during brushing, and have established that preschool children have not attained the manual dexterity that is needed for the maintenance of oral hygiene. In this study we found that only 32.1% of parents reported supervising their preschool children during brushing. The dental caries experienced by participants may be explained by the lack of accompaniment by parents during tooth brushing or unawareness of parents concerning oral hygiene practices. Similarly findings were documented by other studies conducted in sub Saharan African countries [38].

Preschool children who resided in rural areas experienced greater impact of ECC in comparison to those residing in semi-rural areas of Kisarawe district. Similarly findings were documented by other studies [11,39]. However studies emanating from the sub Saharan countries could not establish such an association [40,41]. Inadequate oral health care information and, inefficient oral hygiene practices seem to explain the high levels of ECC among preschool children residing in rural areas.

A low parent schooling level (informal and primary school levels) may indicate inadequate knowledge regarding oral health care, and subsequently a greater number of ECC in preschool children. The present study analyzed both fathers and mother’s schooling level in association with preschool children ECC. The assessment of both parents is necessary as all of them are main caregivers and educators of their children and, this study could not establish association between parent’s levels of education and ECC to preschool children. In contrast, another study reported an association between preschool ECC and a lower level of parents schooling [42].

However, the comparison between the two studies is limited by methodological differences, such as the study design and the method for evaluating preschool ECC. Moreover, the household economic status is associated with the level of the parents' schooling, access to information regarding health, and access to healthcare services.

In terms of socio-demographic factors, the prevalence of preschool children ECC was associated with age and oral hygiene as was previous reported in recent study conducted in Kisarawe [11]. The trend found concerning a greater prevalence of preschool children ECC was associated with an increase in age, and with caries being more common among the five-year-old's, in agreement with findings reported in other studies [34,43]. This result can be explained by the change in the dietary habits and hygiene practices in older children [34].

This study provided initial evidence regarding the association between preschool children dental caries and underweight (WAZ<-2SD) where the dmft score index was negatively and significantly associated with weight-for-age z-score [Coefficient: -0.11 (95% CI: -0.76, -0.11)]. There are several studies documenting on the association between WAZ and change in ECC among preschool children [38,44]. While a lack of association was occasionally reported [45], several studies, mostly in developed countries, demonstrated a negatively and significant relationship [46,47]. On the other hand, other studies argued that WAZ among preschool children would result in increase in ECC increment [38], which was in accordance with the results of this study.

The global information on the association between WHZ and preschool ECC is inconclusive and controversial. Some studies have shown significant associations while others show weak or no relationship between nutritional status and preschool ECC [45]. In the present study, the association between WHZ and preschool ECC were not established. On the contrary a study

conducted in Sub Saharan Africa reported an association between WHZ and dmft score index, and closely linked it with poverty as a main contributory factor [38]. However, in this study the assessment of participant's poverty or socio-economic status was not considered. Further in this study there was no significant association between stunting HAZ and preschool ECC. This can be attributed to the low prevalence of stunting [1.6% (95% CI; 0.9- 2.7 95% CI)] in comparison to the recent reported stunting in the country was [31.8% (95% CI; 30.7-32.9)] [24] a possibility that the children were not subjected to chronic or prolonged food deficiencies.

The relationship between WAZ/HAZ and preschool ECC could also be confounded by other factors. It is worth noting that the bidirectional associations between preschool caries on one hand, and WAZ and HAZ on the other were independent from a number of risk factors related to the development of dental caries such as sugar consumption, oral hygiene and family residence. Unsurprisingly, sugar consumption was associated with change in preschool dental caries, which is consistent with the literatures on the role of sugars in the development of caries [32,48]. This study provides some implications for future studies. Controlling risk factors such as sugary diet appears to promote general health and decrease the prevalence of many chronic diseases in a more efficient and effective way [49,50].

Several limitations could be found in the present study. Firstly, the major limitation of this study relates to the study design and the fact that these results cannot be generalized to the entire population, because the sample calculation was drawn from preschool children enrolled in public schools however it provides baseline data for further studies. Secondly, some potential factors that contributed to ECC, such as poverty and/or socio-economic status were not included in this study. We did not survey the socio-economic status because many parents were not in formal sectors and expressed uncertainty even on the values of fixed assets they possess when we

conducted the pilot investigation. Thirdly, there may have been an unavoidable recall bias from the children's parents in responding to questions. Finally, the present study was a cross-sectional investigation, so causal inference was limited.

Conclusions

This study demonstrated a significant negative relationship between ECC and children anthropometric measures indicated by weight-for-age, and positive relationship with sugar exposure and poor oral hygiene indicated by visible plaque on upper anterior teeth. Furthermore, these findings highlight the significance of ECC as a potential contributing risk factor for children growth indexes.

Acknowledgements

We acknowledge the Muhimbili University of Health and Allied Sciences, the administrative authorities in Kisarawe District, preschool children for their participation, teachers, parents and all of those with whom we had the pleasure to work during this project.

References

1. Lagerweij MD, van Loveren C. Declining Caries Trends: Are We Satisfied? Curr Oral Heal Reports. 2015;2(4):212–217.

2. Anil S, Anand PS. Early childhood caries: Prevalence, risk factors, and prevention. Front Pediatr. 2017;5(157):1–5.

3. Warren JJ, Blanchette D, Dawson D V., Marshall TA, Phipps KR, Starr D, et al. Factors

associated with dental caries in a group of American Indian children at age 36 months. Community Dent Oral Epidemiol. 2016;44(2):154-61.

4. Östberg AL, Skeie MS, Skaare AB EI. Caries increment in young children in Skaraborg, Sweden: associations with parental sociodemography, health habits, and attitudes. Int J Paediatr Dent [Internet]. 2017;27(2):47–55. Available from: doi: 10.1111/ipd.12225. Epub 2016 Jan 30. PMID: 26826705.

5. Weston-Price S, Copley V, Smith H, Davies GM. A multi-variable analysis of four factors affecting caries levels among five-year-old children; deprivation, ethnicity, exposure to fluoridated water and geographic region. Community Dent Health. 2018;35(4):217–22.

6. Finlayson TL, Siefert K, Ismail AI, Sohn W. Psychosocial factors and early childhood caries among low-income African-American children in Detroit. Vol. 35, Community Dentistry and Oral Epidemiology. 2007.

7. UNICEF. The United Nations Children’s Fund Conceptual Framework for the Determinants of Nutritional status. 1990.

8. Masumo R, Bardsen A, Mashoto K, Åstrøm AN. Child- and family impacts of infants’ oral conditions in Tanzania and Uganda- a cross sectional study. BMC Res Notes. 2012;5(538):1–10.

9. Alvarez JO. Nutrition, tooth development, and dental caries. Am J Clin Nutr. 1995;61(2):410S-416S.

10. Çolak H, Dülgergil Ç, Dalli M, Hamidi M. Early childhood caries update: A review of causes, diagnoses, and treatments. J Nat Sci Biol Med. 2013;4(4):29–38.

11. Masumo RM, Ndekero TS, Carneiro LC. Prevalence of dental caries in deciduous teeth and oral health related quality of life among preschool children aged 4-6 years in Kisarawe, Tanzania. BMC Oral Health. 2020;20(1):1–10.

12. Clarke M, Locker D, Berall G, Pencharz P, Kenny DJ, Judd P. Malnourishment in a population of young children with severe early childhood caries. Pediatr Dent. 2006;28(3):254–9.

13. Wasunna D. Nutritional Status of Children Aged 3–5 Years With and Without Severe Early Childhood Caries in New Nyanza Provincial [Internet]. 2012. Available from: http://erepository.uonbi.ac.ke/bitstream/handle/11295/8298/Wasunna _Nutritional Status Of Children Aged 3 – 5 Years With And Without Severe Early Childhood Caries In New Nyanza Provincial General Hospital Kisumu Kenya.pdf

14. Filstrup SL, Briskie D, Da Fonseca M, Lawrence L, Wandera A, Inglehart MR. Early childhood caries and quality of life: Child and parent perspectives. Pediatr Dent. 2003;25:431-40.

15. Dimaisip-Nabuab J, Duijster D, Benzian H, Heinrich-Weltzien R, Homsavath A, Monse B, et al. Nutritional status, dental caries and tooth eruption in children: A longitudinal study in Cambodia, Indonesia and Lao PDR 11 Medical and Health Sciences 1117 Public Health and Health Services 11 Medical and Health Sciences 1105 Dentistry. BMC Pediatr. 2018;18(1):300.

16. Mishu MP, Hobdell M, Khan MH, Hubbard RM, Sabbah W. Relationship between untreated dental caries and weight and height of 6- to 12-year-old primary school children in Bangladesh. Int J Dent. 2013;2013:1–5.

17. Cleaton- Jones MNKPE. Dental Caries in African Preschool Children: Social Factors as Disease Markers. Public Heal Dent. 1998;58(1):7–11.

18. Ghandor. Caries prevalence among 3-5 years old children in Khartoum. J Indian Denatal Assoc JIDA. 1992;163(10):415–7.

19. Kiwanuka N., Astrom A. AT. Dental caries experience and its relationship to social and behavioural factors among 3–5- year- old children in Uganda. Int J Paediatr Dent. 2004;14:336–46.

20. Njoroge NW, Kemoli AM, Gatheche LW. Prevalence and pattern of early childhood caries among 3-5 year olds in Kiambaa, Kenya. East Afr Med J. 2010;87(3):134–7.

21. Rwakatema DS, Ng'ang'a PM. Early childhood caries in Moshi, Tanzania. East Afr Med J. 2010;87(7):304–10.

22. Bailleul FI, Lopes K, Souames M, Azoguy LS, Frelut M BL. Caries experience in a severely obese adolescent population. Int J Paediatr Dent. 2007;17(5):358–63.

23. Tanzania Food and Nutrition Centre. The United Republic of Tanzania Ministry of Health and Social Welfare: Tanzania National Nutrition Survey 2014. 2014.

24. The United Republic of Tanzania TF and NC. Tanzania National Nutrition Survey 2018. 2019.

25. National Bureau of Statistics. 2012 Population and Housing Census. National Bureau of Statistics, The United Republic of Tanzania. Dar Es Salaam; 2012.

26. Mghasse NE, William F. Practices and Challenges in the Provision of Pre-Primary Education in Tanzania. African Res Rev. 2016;10(1):1.

27. Karjalainen S, Söderling E, Sewón L, Lapinleimu H, Simell O. A prospective study on sucrose consumption, visible plaque and caries in children from 3 to 6 years of age. Community Dent Oral Epidemiol. 2001;29:136–42.

28. Pine CM, Pitts NB, Nugent ZJ. British Association for the Study of Community Dentistry (BASCD) guidance on the statistical aspects of training and calibration of examiners for surveys of child dental health. A BASCD coordinated dental epidemiology programme quality standard. Community Dent Health. 1997;14(Suppl 1):18–29.

29. World Health Organization. Design of an oral health survey. In: Oral health surveys : basic methods. 2013.

30. World Health Organization 2006. WHO Child Growth Standards. 2006.

31. Wang Y. CH. Use of Percentiles and Z -Scores in Anthropometry. V. Handbook of Anthropometry: Physical Measures of Human Form in Health and Disease. New York: © Springer Science+Business Media, LLC 2012; 2012. 29–48 p.

32. Petersen PE, Bourgeois D, Ogawa H E-DS and NC. Global burden of oral diseases. Bull World Heal Organ. 2005;83(9):661–9.

33. World Health Organization 2016. Regional Oral Health Strategy 2016 - 2025: Addressing Oral Diseases as part of Noncommunicable Diseases. Regional Committee for Africa. 2016.

34. Musinguzi N, Kemoli A, Okullo I. Prevalence and Treatment Needs for Early Childhood Caries Among 3–5-Year-Old Children From a Rural Community in Uganda. Front Public Heal. 2019;7:1–7.

35. Nobile CG, Fortunato L, Bianco A, Pileggi C, Pavia M. Pattern and severity of early childhood caries in Southern Italy: A preschool-based cross-sectional study. BMC Public Health. 2014;14:206.

36. Baggio S, Abarca M, Bodenmann P, Gehri M, Madrid C. Early childhood caries in Switzerland: A marker of social inequalities. BMC Oral Health. 2015;15(1):1–9.

37. Gao SS, Zhang S, Mei ML, Lo ECM, Chu CH. Caries remineralisation and arresting effect in children by professionally applied fluoride treatment - a systematic review. BMC Oral Health. 2016;16:12.

38. Folayan MO, Arije O, El Tantawi M, Kolawole KA, Obiyan M, Arowolo O, et al. Association between early childhood caries and malnutrition in a sub-urban population in Nigeria. BMC Pediatr. 2019;19(1):19–21.

39. Tadakamadla SK, Tadakamadla J, Tibdewal H, Duraiswamy P, Kulkarni S. Dental caries in relation to socio-behavioral factors of 6-year-old school children of Udaipur district, India. Dent Res J (Isfahan). 2012;9(6):681–687.

40. Mafuvadze BT, Mahachi L, Mafuvadze B. Dental caries and oral health practice among 12 year old school children from low socio-economic status background in Zimbabwe. Pan Afr Med J. 2013;14:164.

41. Varenne B, Petersen PE, Ouattara S. Oral health status of children and adults in urban and rural areas of Burkina Faso, Africa. Int Dent J. 2004;54(2):83–9.

42. Kato H, Tanaka K, Shimizu K, Nagata C, Furukawa S, Arakawa M, et al. Parental occupations , educational levels , and income and prevalence of dental caries in 3-year-old

Japanese children. Env Heal Prev Med. 2017;22(80):1–7.

43. Singh S, Vijayakumar N, Priyadarshini HR, Shobha M. Prevalence of early childhood caries among 3-5 year old pre-schoolers in schools of Marathahalli, Bangalore. Dent Res J (Isfahan). 2012;9(6):710–714.

44. So M, Ellenikiotis YA, Husby HM, Paz CL, Seymour B, Sokal-Gutierrez K. Early childhood dental caries, mouth pain, and malnutrition in the ecuadorian amazon region. Int J Environ Res Public Health. 2017;14(550):1–12.

45. Alves LS, Susin C, Damé-Teixeira N, Maltz M. Overweight and obesity are not associated with dental caries among 12-year-old South Brazilian schoolchildren. Community Dent Oral Epidemiol. 2012;41(3):224–31.

46. Köksal E, Tekçiçek M, Yalçin SS, Tuğrul B, Yalçin S, Pekcan G. Association between anthropometric measurements and dental caries in Turkish school children. Cent Eur J Public Health. 2011;19(3):147–51.

47. Jamelli SR, Rodrigues CS, de Lira PIC. Nutritional status and prevalence of dental caries among 12-year-old children at public schools: A case-control study. Oral Heal Prev Dent. 2010;8:77–84.

48. Gupta P, Gupta N, Pawar A, Birajdar S, Natt A, Singh H. Role of Sugar and Sugar Substitutes in Dental Caries: A Review. ISRN Dent. 2013;29:519421.

49. Maltz M, Jardim JJ, Alves LS. Health promotion and dental caries. Braz Oral Res. 2010;24(Suppl. 1):18–25.

50. Sheiham A, Watt R. The common risk factor approach: a rational basis for promoting oral

health. Community Dent Oral Epidemiol. 2000;28(6):399–406.

Figure
Click here to access/download;Figure;Figure 1.docx

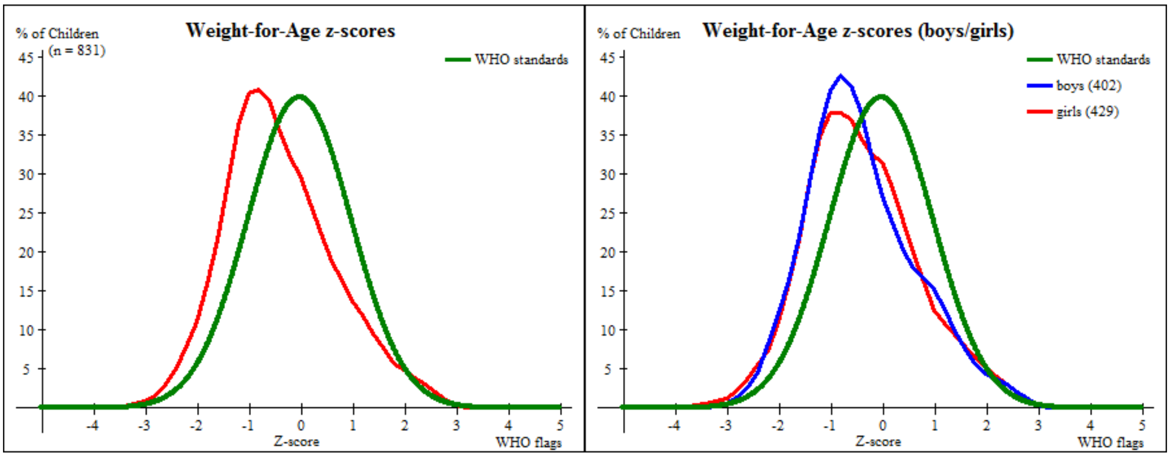

Figure 1: Weight-for-Age z-score (WHO 2006)

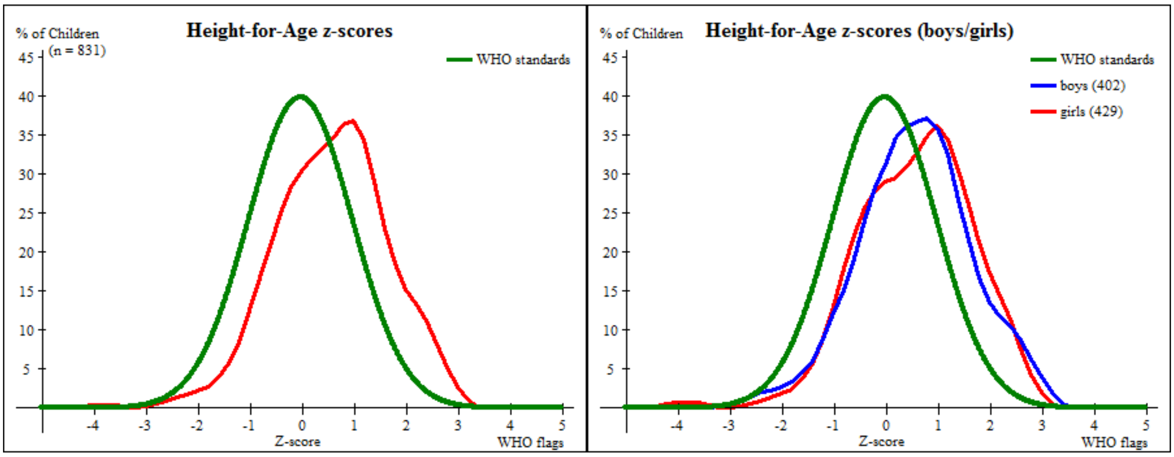

Figure 2: Height-for-Age z-score (WHO 2006)

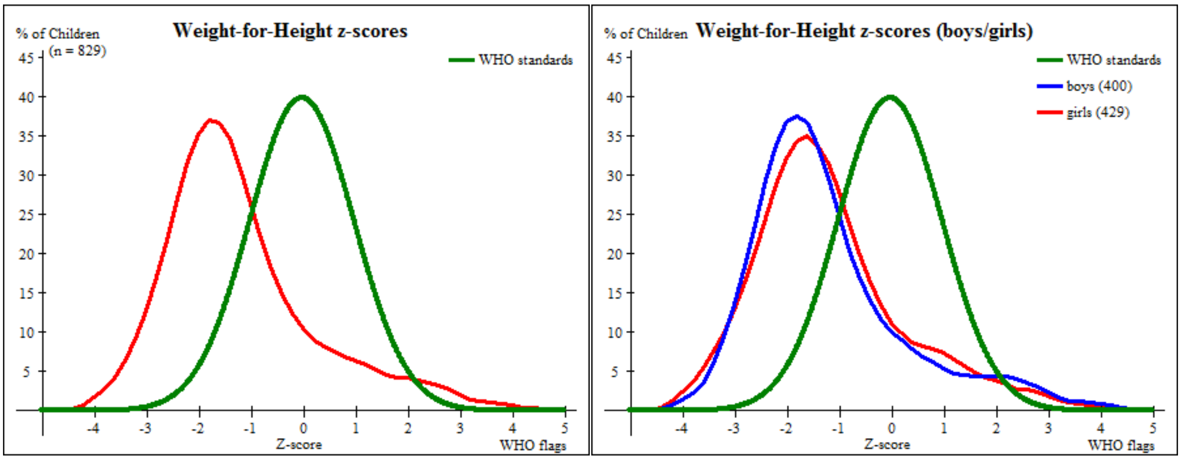

Figure 3: Weight-for-Height z-score (WHO 2006)
