# Supplementary material for: Prevalence of early childhood caries, risk factors and nutritional status among 3-5-year-old preschool children in Kisarawe, Tanzania
Source: PLoS One. 2021 Feb 25;16(2):e0247240. doi: 10.1371/journal.pone.0247240 (PMC7906390; doi:10.1371/journal.pone.0247240)
Supplement: Attachment — Submitted filename: PLOS ONE Manuscript Review.docx [file pone.0247240.s003.docx]

***Prevalence of early childhood caries, risk factors and nutritional status among 3-5-year-old preschool children in Kasarawe, Tanzania.***

**Manuscript** **Review**

Overall ,this is an interesting study on the prevalence of ECC and the risk factors involved, and also investigates the correlation between ECC and anthropometric status in the context of nutrition , diet exposure and oral hygiene status in rural and semi-urban populations in Tanzania. While the approach and efforts are commendable for this relevant topic and study, there are few shortcomings in the methodological approach and in the discussion. In addition , more clarity in describing the results in a more concise and succinct manner is needed. Specific comments follow.

**Minor Suggestions:**

**Abstract**: While the authors give some background information in their abstract , the aim of the study is unclear, could this be rephrased to reflect an overall aim of the study?

**Introduction:** Again, the authors give good background information and context but the significance or rationale for this study is not clearly stated. The authors make reference to a reduction in DMFT in 12-year-old children (line 53) , the age of these children means that they are on average losing their last deciduous teeth, what then is the significance of this evidence when the authors are investigating ECC in 3-5 years old preschool children?

The introduction could be more concise, lines 86-103 are quite repetitive, could the authors condense this information and allude to the similarity in findings and just cite these studies instead?

Could the authors give more context to what actually determines the presence of ECC and perhaps explain the indices used (dmft), this could give better context and relevance of investigating the risk factors associated with ECC.

In the introduction and discussion, the authors allude to other studies to support certain claims however they do not cite these (line 70,71, lines 355-357, lines 380 – 383,) and at times they make claims not backed by any evidence (lines 393 – 395)

**Methods and materials**: The methodological approach could be better explained. Could the background information on the schooling arrangement (lines 115 -118 ) be condensed and made part of the background information in the introduction and instead in this section describe the setting of the data collection.

The information surrounding sample size is unclear – the authors sate in their abstract(line 30) that the study was conducted on 831 children, then also that the sample size of 831 was pre -determined (line 133) yet in the authors also mention only those children who present were chosen to participate(line 136-137).

Could the authors provide the questionnaire used as an appendix rather than writing out the questions in this section (lines 152 -175) and perhaps the clinical examination criterion could be presented in a table instead? (lines183 -186).

**Results**

The results allude to a different sample size of 860 (line 246) and also to parents being the participants, could this be made clearer perhaps in the methods?

Again, the results of the children’s oral health status are not clearly described, are these based on the sample size of 831?

The authors introduce the prevalence of underweight and severe underweight in the discussion but the results of the weight for height scores (line 284 - 287) do not clearly describe or reflect this – what are the indicators for these? These measurements were not clearly explained in the data collection section either (lines 194-204).

**Discussion:**

The authors mention a pilot investigation (line 446), but this was not included in the methods, this should be described and included in the materials and methods section.

The argument on the relevance of weight and age scores is somewhat incoherent, the categorization of these scores is not very clear from the description given, could this perhaps be explained in the results section? (lines 410- 417).

Could the authors have also explored and discussed other significant factors contributing to the prevalence of ECC , such as feeding practices ,familial socioeconomic background and access to dental care. What was the rationale for the authors not considering socio-economic status? (line 425)

The authors mention conveniently choosing the district due to its rural and semi-urban population (line114), however this is not explored further, what are the implications of this? Geographical location is a well-known significant factor contributing to the prevalence of ECC, could the authors consider this ?

**Other comments:**

The authors have not numbered the pages as per the manuscript submission requirements.

The in-text citations are missing in some sections (see previous comment on referencing and citation)

Some grammatical errors can be observed throughout the manuscript (lines 25,55, 123-124, 410- 411, 258,273,276,302,362,373,385)

Abbreviations not defined – dmft (line 36) , 2SD (line 411)
