# Supplementary material for: Prevalence of early childhood caries, risk factors and nutritional status among 3-5-year-old preschool children in Kisarawe, Tanzania
Source: PLoS One. 2021 Feb 25;16(2):e0247240. doi: 10.1371/journal.pone.0247240 (PMC7906390; doi:10.1371/journal.pone.0247240)
Supplement: Attachment — Submitted filename: Response to reviewers.doc [file pone.0247240.s004.doc]

A REBUTTAL LETTER TO ACADEMIC EDITOR AND REVIEWERS

RESPONSE TO COMMENTS FROM THE ACADEMIC EDITOR

ACADEMIC EDITORS COMMENT: *Your manuscript has merit but does not fully meet PLOS ONE’s publication criteria as it currently stands. Therefore, we invite you to submit a revised version of the manuscript that addresses the points raised during the review process.*

**RESPONSE: Thank you for coordinating the review process. We acknowledge the time and effort that you and the reviewers have dedicated on providing invaluable comments and suggestions on this manuscript. We appreciate the opportunity given to submit a revised version of the manuscript following addressing of the points raised during the review process.**

**RESPONSE TO COMMENTS FROM REVIEWER 1**

REVIEWERS COMMENT: *Overall, this is an interesting study on the prevalence of ECC and the risk factors involved, and also investigates the correlation between ECC and anthropometric status in the context of nutrition, diet exposure and oral hygiene status in rural and semi-urban populations in Tanzania. While the approach and efforts are commendable for this relevant topic and study, there are few shortcomings in the methodological approach and in the discussion. In addition, more clarity in describing the results in a more concise and succinct manner is needed.*

**RESPONSE: Thank you for coordinating the review process. We appreciate the time and effort that you and the reviewers have dedicated on providing invaluable comments and suggestions on this manuscript. We thank and appreciate you for commending our efforts towards this study, the methodological approach and discussion have been revised to address the mentioned shortcomings. The result section has also been revised for clarity.**

***Specific comments:***

COMMENT: *In Abstract; while the authors give some background information in their abstract, the aim of the study is unclear, could this be rephrased to reflect an overall aim of the study?*

**RESPONSE: We agree with this comment and the abstract section has been rephrased to clearly reflect the aim of the study.**

COMMENT: *In introduction; again, the authors give good background information and context but the significance or rationale for this study is not clearly stated.*

**RESPONSE: We agree with this comment and the introduction section has been rephrased to show the significance of this study.**

COMMENT: *In introduction; the authors make reference to a reduction in DMFT in 12-year-old children (line 53), the age of these children means that they are on average losing their last deciduous teeth, what then is the significance of this evidence when the authors are investigating ECC in 3-5 years old preschool children?*

**RESPONSE: Thank you very much for pointing this out. We agree with this comment and line 53 has been deleted.**

COMMENT: *The introduction could be more concise, lines 86-103 are quite repetitive, and could the authors condense this information and allude to the similarity in findings and just cite these studies instead?*

**RESPONSE: We appreciate this comment and lines 86-103 have been condensed as suggested.**

COMMENT: *In introduction; could the authors give more context to what actually determines the presence of ECC and perhaps explain the indices used (dmft), this could give better context and relevance of investigating the risk factors associated with ECC.*

**RESPONSE: Thank you very much for pointing this out. We agree with this comment and your suggestions have been incorporated in the text.**

COMMENT: *In the introduction and discussion, the authors allude to other studies to support certain claims however they do not cite these (line 70,71, lines 355-357, lines 380 – 383,) and at times they make claims not backed by any evidence (lines 393 – 395)*

**RESPONSE: Thank you very much for pointing this out. We have addressed the in-text citations and ensure that are cited.**

COMMENT: *In the materials and methods; the methodological approach could be better explained. Could the background information on the schooling arrangement (lines 115 -118) be condensed and made part of the background information in the introduction and instead in this section describe the setting of the data collection.*

**RESPONSE: We agree with this comment and lines 115 -118 have been removed.**

COMMENT: *In the materials and methods; the information surrounding sample size is unclear – the authors state in their abstract (line 30) that the study was conducted on 831 children, then also that the sample size of 831 was pre -determined (line 133) yet in the authors also mention only those children who present were chosen to participate (line 136-137).*

**RESPONSE: Thank you very much for pointing this out. We agree with this comment and have rephrased the information regarding sample size.**

COMMENT: *The results allude to a different sample size of 860 (line 246) and also to parents being the participants, could this be made clearer perhaps in the methods?*

*Again, the results of the children’s oral health status are not clearly described, are these based on the sample size of 831?*

**RESPONSE: We agree with this comment and information regarding sample size has been clarified.**

COMMENT: *The authors introduce the prevalence of underweight and severe underweight in the discussion but the results of the weight for height scores (line 284 - 287) do not clearly describe or reflect this – what are the indicators for these? These measurements were not clearly explained in the data collection section either (lines 194-204).*

**RESPONSE: We agree with this comment and have rephrased the statement regarding the weight for height scores (lines 284 - 287).**

**Furthermore, the information regarding the measurements (reference to lines 194-204) was provided in the statistical analysis section (lines 232- 237).**

COMMENT: *In the discussion; the authors mention a pilot investigation (line 446), but this was not included in the methods, this should be described and included in the materials and methods section.*

**RESPONSE: Thank you very much for pointing this out. We agree with this comment and information on the pilot investigation has been included to the materials and methods section.**

COMMENT: *In the discussion; the argument on the relevance of weight and age scores is somewhat incoherent, the categorization of these scores is not very clear from the description given, could this perhaps be explained in the results section? (Lines 410- 417).*

**RESPONSE: Thank you very much for pointing this out. The description of weight and age scores and its categorization has been included in the result section (refer Table 1).**

COMMENT: *In the discussion; could the authors have also explored and discussed other significant factors contributing to the prevalence of ECC, such as feeding practices, familial socioeconomic background and access to dental care. What was the rationale for the authors not considering socio-economic status? (Line 425)*

**RESPONSE: Thank you for the comment. It would have been interesting to explore these aspects. However, familial socioeconomic background and access to dental care was not part of this study and contributed to the discussed limitation of this study.**

**Furthermore, “What was the rationale for the authors not considering socio-economic status? (Line 425)”. This aspect has been mentioned in the limitation factors of this study. The last paragraph of the discussion section reads “We did not survey the socio-economic status because during the pilot investigation many parents were observed not to be in formal employment and expressed uncertainty in determining value of fixed assets owned”.**

COMMENT: *In the discussion; the authors mention conveniently choosing the district due to its rural and semi-urban population (line114), however this is not explored further, what are the implications of this? Geographical location is a well-known significant factor contributing to the prevalence of ECC, could the authors consider this?*

**RESPONSE: Thank you very much for pointing this out. Differences between the rural and semiurban population have been reported in the Results section line 313-314; and in Table 2 and also in the discussion (line 387 -389)**

**Other comments:**

COMMENT: *The authors have not numbered the pages as per the manuscript submission requirements.*

**RESPONSE: Thank you for this observation. We have inserted pages numbers as per manuscript submission requirements.**

COMMENT: *The in-text citations are missing in some sections (see previous comment on referencing and citation*).

**RESPONSE: Thank you for this observation. We have addressed the in-text citations and ensured that all are cited.**

COMMENT: *Some grammatical errors can be observed throughout the manuscript (lines 25, 55, 123-124, 410- 411, 258,273,276,302,362,373,385)*

**RESPONSE: Thank you for this observation. We have addressed the grammatical errors throughout the manuscript**

COMMENT: *Abbreviations not defined – dmft (line 36), 2SD (line 411)*

**RESPONSE: We have defined dmft and SD. Line 411 has been rephrased and the statement 2SD has been removed.**

**RESPONSE TO COMMENTS FROM REVIEWER 2**

REVIEWER COMMENT: *Line (L) 44 - Besides summarising the findings it is important to include the take home message in the conclusion.*

**RESPONSE: Thank you for the comment. We have added a few sentences regarding take home message in the conclusion.**

REVIEWER COMMENT: *Line (L) 103- It may be worth looking at the research from for example B Monse ...* [*https://pubmed.ncbi.nlm.nih.gov/?term=Monse+B&sort=date*](https://pubmed.ncbi.nlm.nih.gov/?term=Monse+B&sort=date)

**RESPONSE: Thank you very much for pointing this out. We have reviewed the works of Monse B and colleagues and indeed your suggestion was useful.**

REVIEWER COMMENT: *Line (L) 110- The Introduction needs to indicate the potential benefits and translations of the research proposed. This will be important for building the rationale for the study but also to return to in the discussion. The review of literature could be more concise rather than detailing a number of publications individually.*

*The research and publications from the Global Burden of Disease (for e.g. https://pubmed.ncbi.nlm.nih.gov/32122215/) and Lancet series on oral health (for e.g. https://pubmed.ncbi.nlm.nih.gov/31327369/) may assist with making the argument for the study more obvious.*

**RESPONSE: We agree with this comment. We have corrected and revised the introduction to include the rationale of this study and have used the suggested references. We have also rephrased the review of literature to be more concise**

REVIEWER COMMENT: *Line (L) 122- A map may assist an international reader visualise where this is.*

**RESPONSE: Thank you for the suggestion. We have added a map (see Fig 1)**

REVIEWER COMMENT: *Line (L) 139- How was the questionnaire developed? Were questions from validated tools used?*

**RESPONSE: Thank you for the comment. A standard questionnaire was used in this study and the questions were piloted and validated.**

REVIEWER COMMENT: *Line (L) 175- This detailed section could be reduced by referring to a table in the results ... rather than detailing each variable and possible responses ... except where variables have been summarised, for e.g. sugar consumption, this can be detailed here.*

**RESPONSE: Thank you for the comment. The sentences have been rephrased to avoid detailing of each variable and possible responses.**

REVIEWER COMMENT: *Line (L) 213- Any inter-examiner reliability?*

**RESPONSE: Thank you for the comment. This information is included in line 210.**

REVIEWER COMMENT: *Line (L) 248- Is this the general unemployment rate?*

**RESPONSE: Thank you very much for requesting clarification. This is not the general unemployment rate rather it refers to parents who are not formally employed. The sentence has been rephrased for better understanding.**

REVIEWER COMMENT: *Line (L) 261- A table of these findings would be useful to include*.

**RESPONSE: Thank you for the suggestion and it would have been interesting to explore this aspect. However, due to the number of tables included in the manuscript we are of the opinion that description will be acceptable for better understanding.**

REVIEWER COMMENT: *Line (L) 266- Is this figure central to the objectives of the study?*

**RESPONSE: Thank you for the comment. This figure is part of the study objectives and we have modified the caption for better understanding.**

REVIEWER COMMENT: *Line (L) 268- Is this all that is important? Struggled to find this info as it is embedded in the variable column*.

**RESPONSE: Thank you for your comment which we are in agreement with. We have made editions throughout the manuscript.**

REVIEWER COMMENT: *Line (L) 270- I am struggling to understand this table ... there are analyses (findings) in the variable column which is unusual, and not quite certain what the last two columns represent.*

**RESPONSE: Thank you for the observation which we are in agreement with. We have corrected and revised the table.**

REVIEWER COMMENT: *Line (L) 278- Is this central to the study?*

**RESPONSE: Thank you for the valid question. Yes, this figure is part of the study objectives. For better understanding, we have modified the caption.**

REVIEWER COMMENT: *Line (L) 279- Now back to Table ... going between tables and figures and back to same table is not easy for the reader ... deal with main aspects of a table to move to the next set of findings.*

**RESPONSE: We agree with the Reviewer. We have corrected the statements and revised the figures and table.**

REVIEWER COMMENT: *Line (L) 284- Central to study?*

**RESPONSE: Thank you. Yes, this figure is part of the study objectives. For better understanding, we have modified the caption.**

REVIEWER COMMENT: *Line (L) 300- Don't understand this table ... how do I interpret the F-test (df)? RR is? Why different reference groups?*

**RESPONSE: Thank you for the questions. F- test (df) is the results of One-Way ANOVA and RR is results of the Poisson regression and their description is included in the tables, result and discussion sections.**

**Moreover, we preferred to use dmft scores of ECC both as categorical and continuous forms, since using dummy variables runs the risk of losing information, results from logistic regression analyses were checked using Poisson regression with count variables.**

REVIEWER COMMENT: *Line (L) 302- And back to Table 1*

**RESPONSE: We have corrected and revised the text so as to avoid moving back to Table 1.**

REVIEWER COMMENT: *Line (L) 333- Struggling to understand this table, what are the three coefficient columns?*

**RESPONSE: Thank you for requesting clarification. We have corrected and revised the table.**

REVIEWER COMMENT: *Line (L) 345-* Struggling to understand this table, what are the three coefficient columns?

**RESPONSE: We apologize for the struggle in understanding this table. Intention of this table was to further explore the bi-directional association between ECC and the three anthropometric measures (WAZ, WHZ and HAZ).**

**In this table, the three columns represent the WAZ, WHZ and HAZ (as the dependent variables) and ECC (as independent variable).**

REVIEWER COMMENT*: Line (L) 347- Overall the findings are too complicated to appreciate the messages from the study. Keep the analyses simpler to answer the objectives of the study; which may need to be revised.*

**RESPONSE: We are grateful for your comments. We have revised our analyses based on these comments.**

REVIEWER COMMENT*: Line (L) 349- Was this the aim?*

**RESPONSE: We agree with the reviewer and, we have made the necessary revision.**

REVIEWER COMMENT*: Line (L) 449- Discussion much to long and superficial ... needs to focus on main findings, compare and interpret these, but most importantly what is the translation messages (feasible policy and practice impacts)?*

**RESPONSE: Comment appreciated. We have revised the discussion section as suggested.**
